# An Exploratory Analysis of Public Perspectives and Attitudes Towards Radiation in Saudi Arabia

**DOI:** 10.3390/healthcare13192538

**Published:** 2025-10-08

**Authors:** Hanan M. Alzahrani, Fahad Alzahrani, Hala Aljohani, Shouq Albalawi, Shatha Aljurbua, Maisa Elzaki, Walaa Alsharif, Bashair Alhummiany, Awadia Gareeballah, Eman Abdurhman Altay, Tasneem S. A. Elmahdi, Amirah Alsaedi, Manal J. Abdallah, Lamia Ghazi Jamjoom, Bander S. Almutairi

**Affiliations:** 1 Department of Diagnostic Radiology, College of Applied Medical Sciences, Taibah University, Madinah 42353, Saudi Arabiaeaahmed@taibahu.edu.sa (E.A.A.);; 2 Department of Pharmacy Practice, College of Pharmacy, Taibah University, Madinah 30078, Saudi Arabia; 3 Department of Physics, Faculty of Science, Yarmouk University, Irbid 211-63, Jordan; 4 Department of Radiology, Faculty of Medicine, King Abdulaziz University, Jeddah 22254, Saudi Arabia; 5 Radiology Department, King Abdulaziz University Hospital, Jeddah 22252, Saudi Arabia

**Keywords:** awareness, attitudes, radiation, protection, Saudi Arabia

## Abstract

**Aim:** Radiation exposure is a growing public health concern; however, public understanding of its sources, risks, and protective measures remains limited. This study examined familiarity, misconceptions, and attitudes towards both ionising and nonionising radiation among residents of Saudi Arabia, an area that has received limited attention in the literature. **Methods:** A cross-sectional survey was conducted among 888 Saudi residents aged 18 years and above by using a validated online questionnaire. The instrument comprised demographic items, 13 statements assessing radiation familiarity (including knowledge of sources and safety), and 21 items assessing attitudes (including safety practices and willingness to receive further education), all rated on a five-point Likert scale. Descriptive statistics and nonparametric inferential analyses were performed using SPSS v27. **Results:** Participants had a moderate mean familiarity score (3.34 ± 1.16), whereas attitude scores were high (3.56 ± 1.14). Demographic variables, including age, sex, region, and previous training, significantly affected familiarity and attitudes. A medical background and previous radiation education were associated with a higher familiarity level. Nonetheless, most participants expressed a strong interest in acquiring additional knowledge. **Conclusion:** Moderate familiarity with radiation and strong attitudes towards protection among the Saudi public highlight opportunities to strengthen practical safety knowledge. These findings indicate the need for targeted, accessible educational initiatives, particularly through digital platforms, to enhance radiation literacy and support the objectives of Saudi Vision 2030.

## 1. Introduction

Radiation is a broad and often misunderstood topic that includes various forms of energy, each with distinct effects on the human body. It is classified into two categories, namely ionising and nonionising, on the basis of its ability to ionise matter by removing electrons from atoms and molecules [1]. Ionising radiation, which includes X-rays, gamma rays, and particle radiation such as alpha and beta particles, is widely used in medical diagnostics and cancer treatment but poses substantial biological risks. It can damage DNA, induce cellular mutations, and increase cancer risk. By contrast, nonionising radiation lacks sufficient energy to ionise atoms and primarily exerts a heating effect. Examples of nonionising radiation include solar radiation, radio waves, and microwaves. While generally considered less harmful, excessive exposure to certain forms of nonionising radiation, including ultraviolet (UV) rays from the sun, can still cause adverse effects, including skin burns and an increased cancer risk [2,3,4].

Radiation protection is essential to reduce the harmful health effects of radiation exposure. As individuals are increasingly exposed to radiation in daily life through medical imaging, electronic devices, and environmental UV rays, adopting protective measures has become a public health priority. According to the International Commission on Radiological Protection (ICRP), the recommended annual dose limit is 1 millisievert (mSv) for the public. For occupationally exposed workers, the recommended annual dose limit is 20 mSv per year, averaged over 5 years, with no single year exceeding 50 mSv [5]. Practical measures, such as applying broad-spectrum sunscreen, wearing protective clothing, reducing unnecessary medical imaging, and following safety guidelines for electronic devices, can substantially reduce exposure. Public education is vital in equipping individuals with the knowledge to make informed decisions and mitigate health risks associated with both ionising and nonionising radiation [6,7]. Adhering to radiation safety guidelines on a daily basis not only protects individuals but also supports broader societal efforts to maintain a safe radiation environment.

Public understanding of radiation and its sources is crucial for encouraging safe practices and reducing unnecessary exposure. However, familiarity levels vary considerably among countries. A cross-sectional study conducted in Saudi Arabia determined that most participants incorrectly believed that chest X-rays and computed tomography (CT) scans deliver equal doses of radiation, highlighting substantial gaps in public knowledge [8]. Such misconceptions may result in either underestimating or overestimating radiation risks, potentially affecting medical decision-making and personal behaviour. By contrast, countries such as Finland and Norway have established comprehensive radiation protection systems along with proactive public education programmes. The Radiation and Nuclear Safety Authority in Finland and the Norwegian Radiation and Nuclear Safety Authority both actively monitor radiation levels and run nationwide outreach campaigns to inform the public about radiation risks and safety measures [9,10]. These strategies have improved radiation literacy and compliance with safety protocols. Thus, such similar initiatives must be implemented in countries with lower public familiarity. In particular, the Saudi Vision 2030 blueprint, launched in 2016, aims to advance multiple sectors, including education, and has substantial potential to enhance public awareness of radiation among Saudi residents [11].

The present study examined public perceptions and common misconceptions regarding both ionising and nonionising radiation in the Saudi population and identified key knowledge gaps that can inform the development of targeted educational strategies and effective public awareness campaigns in the future.

## 2. Materials and Methods

### 2.1. Ethical Approval

This study was approved by the Ethics and Scientific Research Committee of the Faculty of Applied Medical Sciences (reference number: 2025/203/303 RAD). All participants were provided with clear information on the study, informed that participation was voluntary, and advised of their right to withdraw at any time without consequence. Informed consent was electronically obtained before survey participation. Data were collected from all administrative regions of Saudi Arabia between October 2024 and March 2025.

### 2.2. Study Design and Settings

The questionnaire (see Appendix A for details) was designed to assess familiarity with and behavioural practices regarding radiation among residents aged 18 years and above, and it comprised three sections. Demographic information collected from respondents included age, sex, education level, place of residence, nationality, region, community, occupation (categorised as medical or nonmedical), salary, and interest in learning about radiation. Participants rated their confidence in their understanding of radiation sources and safety (13 items) as well as their attitudes towards radiation, including safety practices and willingness to receive education (21 items), on a 5-point Likert scale with endpoints ranging from 1 (not at all confident) to 5 (highly confident). The total scale range of four units was divided into five equal intervals of 0.80 to facilitate interpretation, allowing responses to be categorised into five levels: low (1.00–1.80), moderately low (1.81–2.60), moderate (2.61–3.40), moderately high (3.41–4.20), and high (4.21–5.00) [12]. In addition, five questions assessed participants’ understanding of radiation sources and risks, covering previous education or training, information sources, experience with medical imaging, sun exposure practices, and familiarity with various radiation sources.

### 2.3. Population, Sample, and Sample Size

This study included Saudi residents aged 18 years and above. An infinite population size was assumed for the sample size calculation. The minimum required sample size was calculated to be 384 participants by using a 95% confidence interval and a 5% margin of error. The following standard formula was used:

$$n=\frac{z^{2}\cdot \hat{p}\;(1-\hat{p})}{\varepsilon^{2}}$$

where *n* is the required sample size, *z* represents the z-score for the desired confidence level, $\hat{p}$ denotes the estimated population proportion, and ε denotes the margin of error [13]

### 2.4. Validity and Reliability

A multistep approach was adopted to ensure the validity and reliability of the questionnaire. Content validity was established through expert review by specialists in radiology and survey methodology who assessed the clarity, relevance, and comprehensiveness of the items. A pilot study was then conducted with a subset of the target population (n = 10) to evaluate internal consistency and refine any ambiguous questions. Reliability was measured using Cronbach’s alpha, with a threshold of 0.70 considered acceptable for internal consistency [14].

### 2.5. Statistical Analysis

Data collected using Google Forms were transferred to an Excel spreadsheet and coded for statistical analysis. All statistical analyses were conducted using SPSS Statistics version 27 (IBM Corp., Armonk, NY, USA). Descriptive statistics, including means, standard deviations, total scores, frequencies, and percentages, were calculated. Because the data were found to be nonnormally distributed by using Kolmogorov–Smirnov and Shapiro–Wilk tests, nonparametric statistical methods were applied. The Mann–Whitney U test was used to examine associations between attitudes and familiarity with radiation and binary demographic variables, while the Kruskal–Wallis test was applied for demographic variables with more than two categories. The significance level (α) for all statistical tests was set at 0.05, and two-tailed tests were employed throughout.

## 3. Results

The items included in the study questionnaire demonstrated strong internal consistency, with an overall Cronbach’s alpha of 96.2%. Cronbach’s alpha values were also calculated for each domain separately, yielding 94.2% for the 13 items on radiation familiarity and 96.5% for the 21 items on perceptions and attitudes towards radiation exposure.

### 3.1. Participant Characteristics

A total of 888 participants were included to enhance statistical power and the generalisability of the findings. The largest age group was 18–24 years (28.9%), and the sample comprised a higher proportion of women (75.1%) and Saudi nationals (94.6%). Most participants (58.6%) held a bachelor’s degree, whereas 9.8% had postgraduate qualifications. The majority (96.7%) resided in urban areas, and 86.8% were from nonmedical backgrounds. Geographically, most respondents (72.1%) were from the Western region. In terms of health-related experiences, 75.5% had undergone medical imaging at least once in the past 2 years, whereas only 23.9% reported actively avoiding direct exposure during peak radiation hours. Further demographic details are presented in Table 1.

Table 2 highlights key differences in familiarity and attitude scores across demographic groups. Awareness was highest among older participants (>60 years, *p* = 0.13), males (*p* = 0.03), medical professionals (*p* < 0.01), and those with prior radiation training (*p* < 0.01), while attitudes were more positive among females (*p* = 0.02) and residents of the Southern and Western regions (*p* = 0.08). Significant differences were also observed for employment status, interest in learning, and sun exposure (*p* ranging from <0.01 to 0.02). These results suggest targeted educational efforts are needed for younger, non-medical, urban populations and those without prior training to enhance radiation literacy and preventive practices across the Saudi population.

### 3.2. Insights into Radiation Sources and Risk Assessment

**Figure 1:**
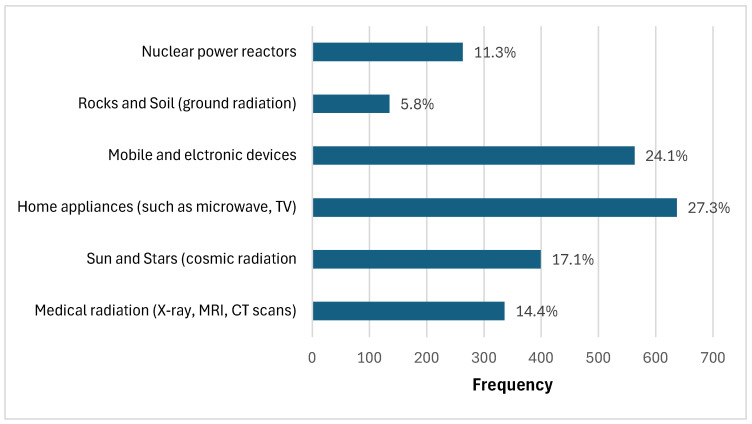
Public perceptions of radiation sources. Most respondents identified home appliances and mobile devices as major sources.

The most frequently recognised radiation sources were home appliances (27.3%), mobile devices (24.1%), and cosmic radiation from the sun and stars (17.1%). Medical imaging procedures were identified by 14.4% of participants, whereas fewer recognised nuclear power reactors (11.3%) and natural ground radiation (5.8%) as sources of radiation (Figure 1).

**Figure 2:**
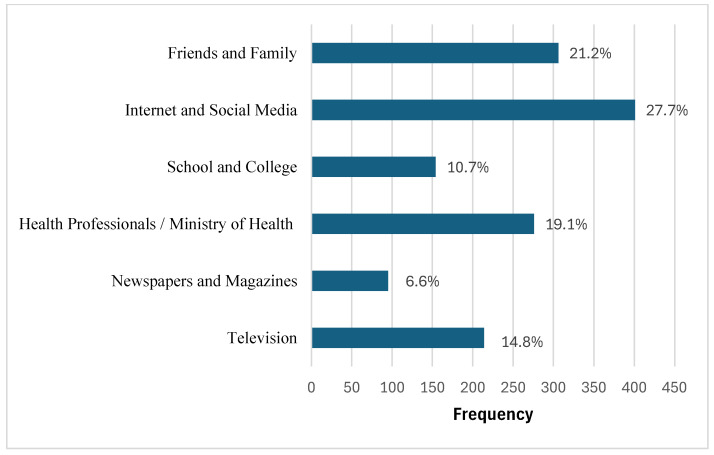
Sources of radiation information reported by participants. The Internet and social media were the most cited sources.

As depicted in Figure 2, the most commonly reported information sources on radiation protection were the Internet and social media (27.7%), followed by friends and family (21.2%), and health professionals or the Ministry of Health (19.1%). Television, schools, and colleges were cited less frequently.

The results, based on a 5-point Likert scale (maximum score = 5) for 13 items, indicated varying levels of familiarity and understanding. Participants expressed strong agreement on the importance of public radiation education (3.58 ± 1.13), placing it in the high category. By contrast, familiarity with safety methods for consumer products emitting radiation scored lowest (3.14 ± 1.14), falling within the medium category. The overall mean score across all items was 3.34 ± 1.16, indicating a moderate level of familiarity. These findings suggest that although the importance of radiation education is widely recognised, practical and detailed knowledge remains limited, indicating the need for targeted educational initiatives.

### 3.3. Participants’ Attitudes Towards Radiation Exposure

The results, based on a 21-item questionnaire using a 5-point Likert scale, produced an overall mean score of 3.56 ± 1.14, placing the general response level in the high category. The highest-rated item, ‘General education on radiation sources is necessary for the safety of society’ (4.03 ± 1.09), fell into the very high category, reflecting strong support for public education on radiation. By contrast, the lowest-rated statement, ‘The media often exaggerates the risks of exposure to radiation’ (2.92 ± 1.12), received only moderate agreement, suggesting mixed perceptions regarding media coverage. Overall, the findings indicate a relatively well-informed and cautious attitude towards radiation, with a particular focus on education and safety awareness.

Several demographic factors were significantly associated with participants’ familiarity and attitudes. Age was associated with both familiarity and attitude scores, with participants over 60 years having the highest scores. Men exhibited higher familiarity (*p* = 0.03), whereas women displayed more favourable attitudes towards radiation exposure (*p* = 0.02). Participants from the Western and Southern regions had higher familiarity scores (*p* = 0.02). Those with a medical background, employed individuals, and participants who had received education or training in radiation protection demonstrated significantly higher familiarity (*p* < 0.01). In addition, participants who expressed an interest in learning about radiation protection had higher attitude scores (*p* < 0.01). By contrast, no significant association was observed between education level or nationality and radiation familiarity or attitudes. Participants with longer exposure to sunlight also had higher familiarity scores (*p* = 0.02).

## 4. Discussion

Exposure to ionising radiation, even at low doses, poses potential health risks, including cellular damage, an increased cancer risk, and other long-term biological effects [15,16]. Although radiation is widely used in medical imaging, industry, and consumer products, inadequate awareness and poor safety practices can increase the risk of unnecessary exposure, particularly among the public. Understanding public knowledge, perceptions, and behaviours regarding radiation is therefore essential for informing educational initiatives and regulatory strategies. This study explored how individuals perceive and respond to radiation-related problems in a diverse population sample in Saudi Arabia.

In the current study, participants most frequently identified everyday items such as mobile devices and home appliances as sources of radiation, whereas medical imaging procedures and natural sources were less commonly recognised (Figure 1). This finding is consistent with that of a recent Saudi study indicating that over two-thirds of respondents were unaware of natural radiation sources and 41.5% mistakenly believed that chest X-rays and CT scans emit equal doses [8]. This pattern reflects global trends, in which the public tends to underestimate the risks associated with medical imaging and natural radiation exposure [17,18,19]. Furthermore, a comprehensive review highlighted ongoing confusion between ionising and nonionising radiation as well as a tendency to regard all radiation as equally harmful, a misconception rooted in inadequate education and media distortion [20].

Despite the relatively high level of education in the sample (68.4% had a bachelor’s degree or higher), a substantial proportion (79.5%) had not received formal education or training in radiation protection (Table 1). This gap is in line with the findings of previous studies indicating that radiation safety knowledge remains limited even among well-educated populations without targeted training [21]. One study reported that, even among healthcare workers, only 40% had sufficient knowledge of radiation hazards and protective measures [22]. Similar results were observed in studies conducted in the United Arab Emirates among nursing students [23]. These findings highlight the urgent need for effective radiation protection education and training, delivered by specialists in a clear and accessible format. Dissemination through the Internet and social media is particularly crucial because these platforms were the most frequently cited sources of radiation-related information by participants in the present study (Figure 2).

Participants generally exhibited cautious attitudes towards radiation exposure, as reflected by the overall high mean score (3.56 ± 1.14). There was strong support for public education on safety (mean = 3.99 ± 1.1), stricter regulations (mean = 3.93 ± 1.09), and awareness of UV radiation risks (mean = 3.91 ± 1.1). Agreement was particularly high regarding the importance of educating the public about radiation sources (mean = 4.03 ± 1.1). However, protective behaviours were less consistently reported; for example, regularly inspecting radiation-emitting devices received one of the lowest scores (mean = 3.14 ± 1.23). This gap between awareness and behaviour has also been observed in previous studies [21,24]. It may result from limited familiarity with radiation sources and associated hazards because the public often relies on unreliable information sources [25]. Furthermore, the physical invisibility of radiation contributes to uncertainty; as it cannot be perceived directly, individuals may doubt the effectiveness of preventive measures, reducing their motivation to act.

Given that the sample was largely female (75%), urban (96%), and from the Western region (96.7%), the findings may reflect the perspectives of this subgroup and should be interpreted with caution when generalising to the broader population. Demographic factors affected both familiarity and attitudes. Older participants (>60 years) and men demonstrated higher familiarity scores, whereas women exhibited more favourable attitudes. In general, men perceived risks as significantly lower than women, which may account for the more cautious and positive attitudes towards radiation observed among women [8,26]. Psychologically, older individuals may display increased concern regarding health-related risks, particularly when such risks are personally relevant or informed by previous experiences [19]. Regional differences were also noted, with participants from the Western and Southern regions reporting higher familiarity levels, whereas those from the Eastern region demonstrated more positive attitudes. Environmental and social factors, including social status, feelings of alienation, and levels of trust, play a significant role in shaping individuals’ perceptions and acceptance of risks [26,27].

The current study has certain limitations, most notably its relatively small sample size. Although the study provided an exploratory analysis representative of the entire country, the majority of respondents were women or from the Western region, which limits representation from other geographical areas. Thus, the generalisability of the findings to the wider Saudi population may be constrained. Nevertheless, this limitation indicates a crucial observation: even within a highly educated cohort, there is a considerable need for improved public education on radiation exposure and preventive measures in Saudi Arabia. This need is particularly relevant in the context of the Saudi Vision 2030 blueprint, launched in 2016, which seeks to advance multiple sectors, including education, and has significant potential to enhance public awareness of radiation across the kingdom. It is recommended that future studies use stratified sampling and weighting to improve representativeness.

## 5. Conclusions

This study determined that participants had a moderate level of familiarity with radiation and its risks, particularly in relation to medical procedures and radiation protection measures, along with a high level of positive attitude. However, notable knowledge gaps were identified regarding safety practices and radiation sources. To address these gaps, targeted educational programmes are recommended to enhance public awareness and encourage safe practices related to radiation exposure, with particular emphasis on medical imaging procedures and sun safety measures.

## Figures and Tables

**Table 1:** Participants’ demographic characteristics.

Characteristics	n	%
Age (years)		
18–24	257	28.9
25–34	142	16.0
35–44	157	17.7
45–54	178	20.0
55–60	81	9.1
>60	57	6.4
Sex		
Male	221	24.9
Female	667	75.1
Education level		
Primary/elementary education	17	1.9
High school	169	19.0
Diploma	95	10.7
Bachelor’s (BSc) degree	520	58.6
Postgraduate (MSc, PhD) degree	87	9.8
Nationality		
Saudi	840	94.6
Non-Saudi	48	5.4
Geographic area		
Central region	152	17.1
Western region	640	72.1
Eastern region	18	2.0
Southern region	31	3.5
Northern region	47	5.3
Place of residence		
Rural	29	3.3
Urban	859	96.7
Professional background		
Medical	117	13.2
Nonmedical	771	86.8
Employment Status		
Student	231	26.0
Unemployed	111	12.5
Employed	382	43.0
Free work	33	3.7
Retired	131	14.8
Received any education or training on radiation protection		
No	706	79.5
Yes	109	12.3
Unsure	73	8.2
Interested in learning about radiation protection		
No	25	2.8
Yes	786	88.5
Unsure	77	8.7
Time spent in direct sunlight (10 AM to 4 PM) without protection		
<30 min	333	37.5
30–60 min	120	13.5
>1 h	96	10.8
Avoid direct sunlight	212	23.9
Unsure	127	14.3
Times you have had medical imaging (e.g., X-rays, CT, and MRI) in the past 2 years		
None	175	19.7
Once	151	17.0
Twice	168	18.9
3–5 times	211	23.8
>5 times	140	15.8
Unsure	43	4.8

**Table 2:** Association between participants’ demographic characteristics and familiarity and attitude scores (n = 888).

Characteristics	Awareness Score		Attitude Score	
	Mean ± SD	*p*-Value	Mean ± SD	*p*-Value
Age				
18–24	3.40 ± 0.92	0.13	3.44 ± 0.78	0.01 *
25–34	3.38 ± 0.89		3.49 ± 0.77	
35–44	3.33 ± 0.96		3.67 ± 0.78	
45–54	3.26 ± 0.79		3.64 ± 0.74	
55–60	3.17 ± 0.92		3.44 ± 0.85	
>60	3.82 ± 0.81		3.74 ± 0.70	
Sex				
Male	3.43 ± 1.00	0.03 *	3.45 ± 0.89	0.02 *
Female	3.31 ± 0.85		3.60 ± 0.75	
Education level				
Primary/Elementary	3.54 ± 0.98	0.96	3.70 ± 0.63	0.20
High School	3.35 ± 1.00		3.48 ± 0.90	
Diploma	3.38 ± 0.94		3.64 ± 0.79	
Bachelor’s (BSc)	3.31 ± 0.85		3.57 ± 0.76	
Postgraduate (MSc, PhD)	3.30 ± 0.89		3.46 ± 0.75	
Nationality				
Saudi	3.34 ± 0.89	0.83	3.65 ± 0.79	0.39
Non-Saudi	3.33 ± 0.85		3.52 ± 0.63	
Geographical area				
Central region	3.23 ± 0.85	0.02 *	3.50 ± 0.73	0.08
Eastern region	3.33 ± 0.89		3.57 ± 0.79	
Western region	3.75 ± 0.50		3.65 ± 0.71	
Southern region	3.60 ± 1.12		3.77 ± 0.95	
Northern region	3.29 ± 1.01		3.34 ± 0.83	
Place of residence				
Rural	3.40 ± 1.23	0.36	3.78 ± 0.72	0.07
Urban	3.33 ± 0.88		3.55 ± 0.79	
Professional background				
Medical	3.62 ± 0.99	<0.01 *	3.59 ± 0.87	0.45
Non-medical	3.29 ± 1.00		3.56 ± 0.77	
Employment status				
Student	3.43 ± 0.92	<0.01 *	3.44 ± 0.80	<0.01 *
Unemployed	3.05 ± 0.93		3.39 ± 0.83	
Employed	3.39 ± 0.86		3.64 ± 0.75	
Free work	3.07 ± 0.92		3.41 ± 0.85	
Retired	3.27 ± 0.86		3.63 ± 0.79	
Received any education or training on radiation protection				
No	3.23 ± 0.86	<0.01 *	3.53 ± 0.75	0.55
Yes	3.85 ± 1.00		3.64 ± 1.02	
Unsure	3.48 ± 0.76		3.57 ± 0.75	
Interested in learning about radiation protection				
No	3.27 ± 0.90	<0.01 *	3.24 ± 0.74	<0.01 *
Yes	3.36 ± 0.89		3.60 ± 0.78	
Unsure	2.96 ± 0.80		3.16 ± 0.80	
Time spent in direct sunlight (10 AM to 4 PM) without protection				
<30 min	3.34 ± 0.86	0.02 *	3.53 ± 0.74	0.38
30–60 min	3.42 ± 0.98		3.56 ± 0.84	
>1 h	3.52 ± 1.01		3.55 ± 0.93	
Avoid direct sunlight	3.22 ± 0.90		3.59 ± 0.79	
Unsure	3.26 ± 0.76		3.54 ± 0.72	
Times you have had medical imaging (e.g., X-rays, CT, and MRI) in the past 2 years				
None	3.36 ± 0.93	0.13	3.54 ± 0.80	0.10
Once	3.21 ± 0.88		3.50 ± 0.82	
Twice	3.37 ± 0.97		3.56 ± 0.83	
3–5 times	3.25 ± 0.86		3.55 ± 0.73	
>5 times	3.49 ± 0.81		3.67 ± 0.76	
Unsure	3.33 ± 0.85		3.32 ± 0.80	

* Significant *p*-value < 0.05.

## Data Availability

The data that support the findings of this study are available from the corresponding author upon reasonable request.

## Appendix A

The research questionnaire:

Section 1: Demographic Information

1. Age:
2. Sex:
3. Level of Education:
4. The current place of residence:
5. Nationality
6. Region:
7. Community:
8. Professional:
9. Specialisation/field: Medical or Non-medical
10. Are you interested in learning more about how to protect yourself from radiation?

Section 2: General awareness of radiation sources and risk

11. Have you ever received any education or training on radiation protection, including online training courses?
12. Where do you get most of your information about radiation and its effects? (select all that applies):
13. How many times have you undergone diagnostic medical imaging (such as dental X-rays, CT scan, MRI, etc.) in the past two years?
14. How long will you spend in direct sunlight (in the peak time 10 am–4 pm) without using any protection (such as sunscreen, hats, sunglasses, … etc.)?
15. What radiation sources do you know? (Select all that apply):
  ◦ Medical radiation (x-ray, MRI, CT)
  ◦ The sun and stars (Cosmic radiation)
  ◦ Household appliances (e.g., microwaves, TV)
  ◦ Mobile devices
  ◦ The rocks and soil (Terrestrial radiation)
  ◦ Nuclear power reactors
  ◦ Other

Below are some statements related to awareness about radiation. Please indicate your level of agreement (strongly agree, agree, neutral, disagree, strongly disagree) with these statements:

1. I am familiar with the term “radiation”
2. I am aware of the different sources of radiation in everyday life
3. I am aware of the concept of radiation protection
4. I am familiar with the safety measures related to radiation in medical fields.
5. I am aware of the potential risks of radiation exposure in my daily life
6. I am familiar with the use of radiation in various industries (e.g., hospitals, airports).
7. I feel knowledgeable about the safety of consumer products that may emit radiation (e.g., Wi-Fi, cell phones).
8. I understand the importance of public awareness regarding radiation sources and protection
9. I regularly take precautions to minimise my exposure to unnecessary radiation.
10. I can notice any changes or signs of skin damage due to radiation exposure from the sun
11. I know the warning symbol for the presence of radiation in the hospital
12. I know how to protect myself from medical imaging (such as using lead shielding or following instructions to avoid repetition)
13. I believe I can distinguish between imaging modalities in the hospitals like ultrasound/MRI and X-ray/CT

Section 3: Feelings and behaviours towards exposure to radiation

Below are some statements related to feelings and practices regarding radiation. Please indicate your level of agreement (strongly agree, agree, neutral, disagree, strongly disagree) with these statements:

1. The media often exaggerates the risks of exposure to radiation.
2. Sunscreen should be used daily, regardless of the weather.
3. It is important for me to use sunglasses when going outdoors.
4. I try hard to stay in the shadows when I am outside.
5. I prefer to cover most of my body when I am outdoors during the day.
6. It is important to limit direct sun exposure to reduce the risk of skin cancer. (I believe that there is a relationship between radiation and cancer).
7. People should be more aware of the UV radiation risks associated with sun exposure.
8. I feel comfortable when undergoing medical procedures that include X-ray imaging when necessary.
9. I consult my doctor before repeating X-rays.
10. Advancements in technology have made X-ray procedures much safer than in the past.
11. Sufficient information about safety from radiation must be provided by professionals in the field of healthcare.
12. The Healthcare Organisations must regulate the use of radiation in various industries more strictly.
13. General education on radiation sources is necessary for the safety of society.
14. I avoid putting the mobile phone near my head for long periods.
15. I am extinguishing the unused electronic devices to reduce radiological emissions.
16. I support spreading awareness about the risks of radiation and how to prevent it.
17. I avoid accompanying patients inside the radiology room if not necessary
18. I regularly inspect devices that emit radiation (such as mobile phones and microwaves) to ensure their safety.
19. I am concerned about the possible health risks associated with radiation exposure.
20. I read the instructions or warnings related to radiation before using electronic devices.
21. I prefer using lower-radiation alternatives to electronic devices when available.
